# Anomalous Origin of Left Anterior Descending Artery and Left Circumflex Artery from Right Coronary Sinus with Malignant Left Anterior Descending Artery Course: Role of Coronary CT Angiography Derived Fractional Flow Reserve in Decision Making

**DOI:** 10.7759/cureus.3220

**Published:** 2018-08-28

**Authors:** Hassan Tahir, Sajjad Ahmad, Muhammad U Awan, Bassam Omar, Joey Glass, Jason Cole

**Affiliations:** 1 Cardiology, USA Medical Center, Mobile, USA; 2 Cardiology, University of South Alabama, Mobile, USA; 3 Director Cardiac Ct, Cardiology Associates, Mobile, USA; 4 Medical Director, Cardiology Associates, Mobile, USA

**Keywords:** coronary anomalies, coronary ct angiogram fractional flow reserve, sudden cardiac death

## Abstract

Congenital coronary anomalies are uncommon and are mostly asymptomatic; however, patients may have symptoms depending on the origin and course of anomalous artery. Very rarely, coronary anomalies can also lead to life-threatening complications especially in young athletes. A malignant course of the left main (LM) or left anterior descending (LAD) artery between aorta and pulmonary artery is considered the most significant risk factor for such complications. Various noninvasive tests are available to evaluate myocardial ischemia due to anomalous coronary artery. Coronary computed tomography (CT) angiogram derived fractional flow reserve (CT-FFR) is a noninvasive diagnostic test which has shown promising results in the hemodynamic assessment of obstructive coronary artery disease. However, its role in coronary anomalies has not been studied. We present a case of a 22-year-old male who presented with atypical chest pain and was found to have anomalous origin of left anterior descending (LAD) artery and left circumflex (LCX) artery from right coronary sinus. LAD had a malignant course for which CT-FFR was done which was hemodynamically nonsignificant. The decision was made to manage the patient conservatively.

## Introduction

Coronary anomalies are incidentally found during invasive and noninvasive coronary imaging and are mostly asymptomatic due to benign course of anomalous coronary artery. However, in rare cases, anomalous coronary artery can have a malignant course which can lead to myocardial ischemia, malignant arrhythmias, or sudden cardiac death [1].

## Case presentation

**Figure 1:**
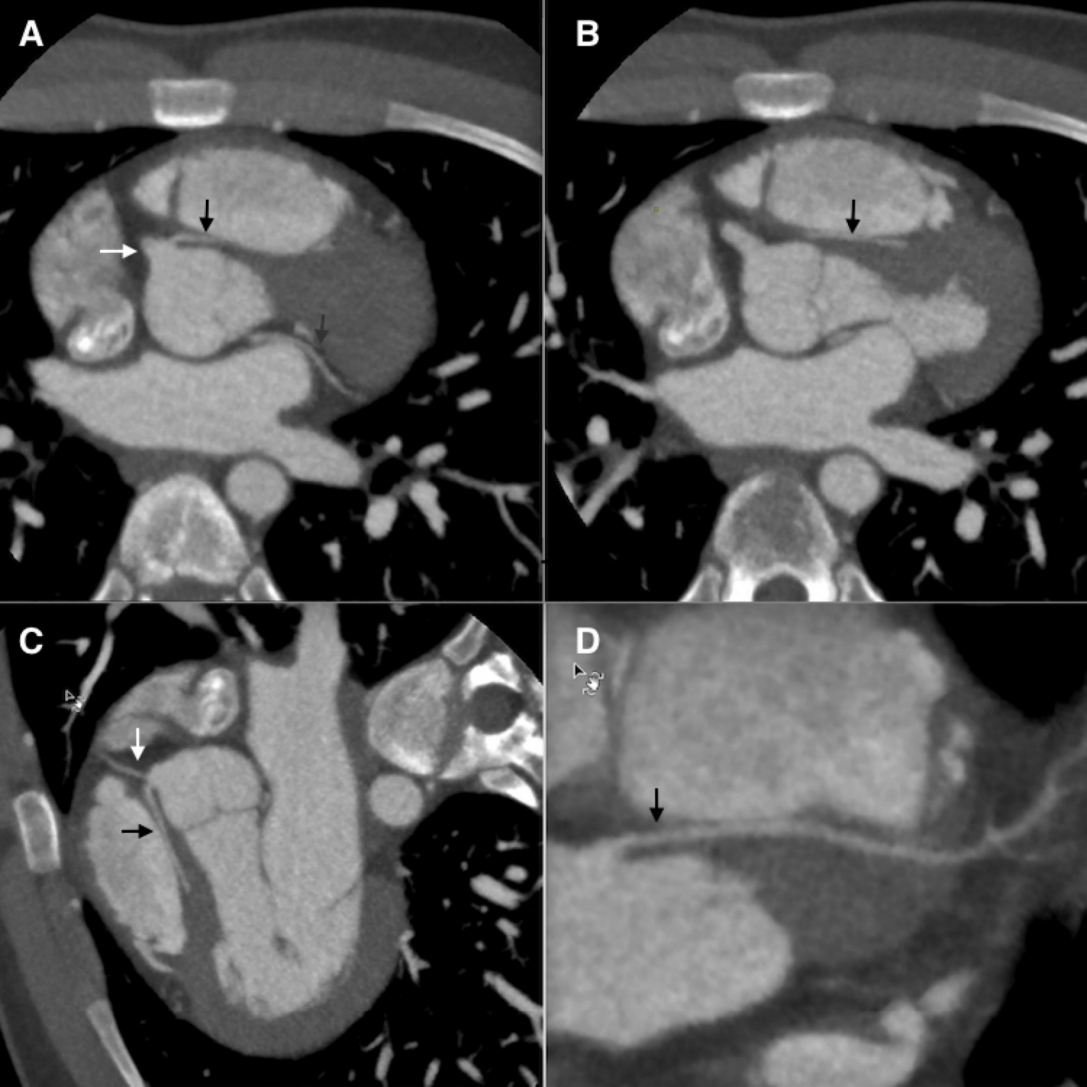
(A) Axial image obtained by 64-slice multidetector computed tomography (MDCT) showing origin of large dominant right coronary artery (white arrow) and anomalous origin of small left anterior descending artery (black arrow) from the right coronary sinus. Small left circumflex artery (gray arrow) is also seen in the image. No plaque was seen in any of three vessels. (B) Anomalous left anterior descending artery (black arrow) seen coursing between ascending aorta and pulmonary trunk. (C) MDCT maximal intensity projection demonstrating anomalous origin and malignant course of left anterior descending artery (black arrow). Also notice origin of small left circumflex artery (white arrow) from right coronary sinus. (D) MDCT image demonstrating a multiplanar reconstruction of the entire anomalous left anterior descending artery (black arrow).

**Figure 2:**
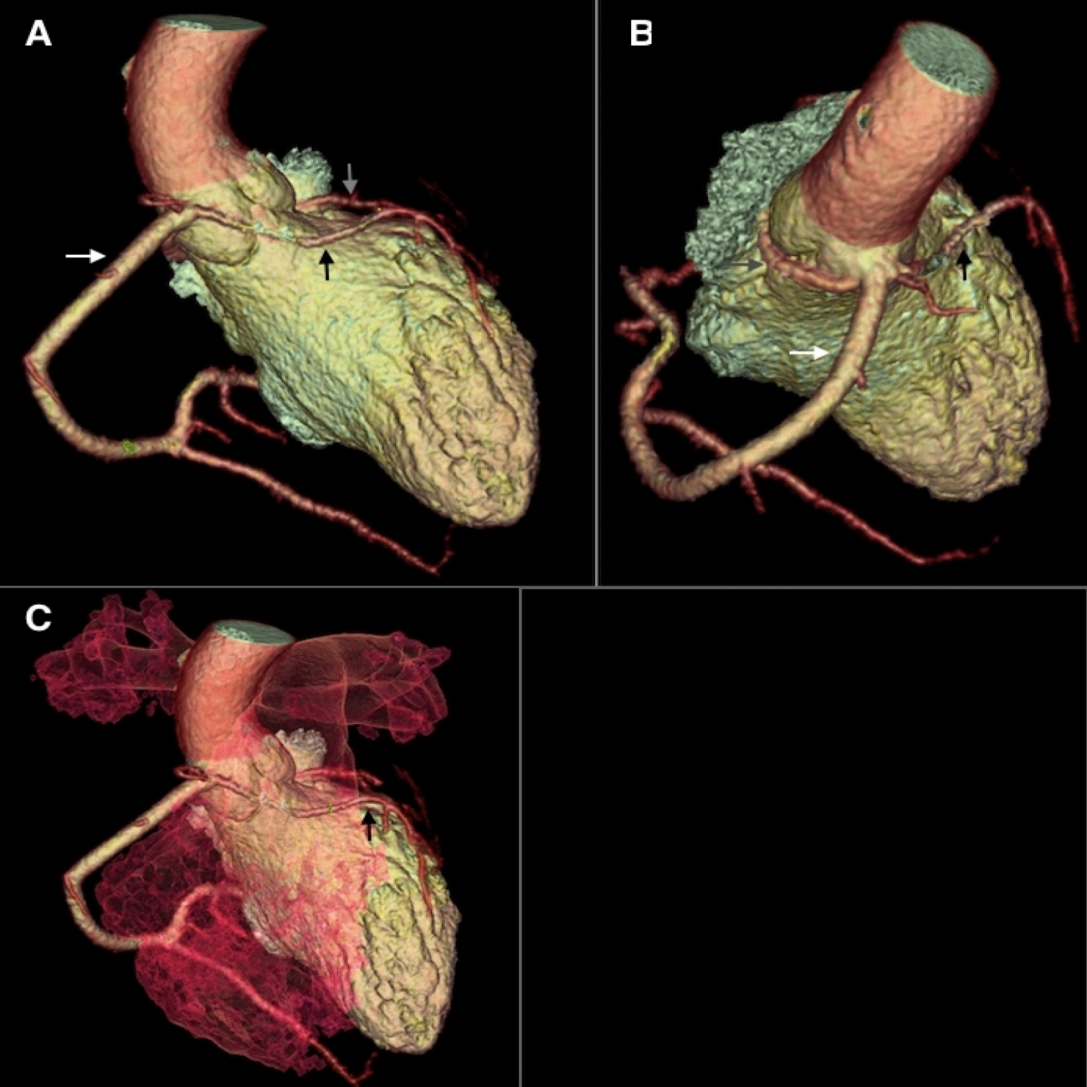
(A) A volume rendered 3D computed tomography (CT) angiogram (anterior view) showing a large dominant right coronary artery (white arrow) arising from right coronary sinus and supplying most area of the myocardium of heart. The right coronary artery measures about 5 mm proximally. Left anterior descending artery (black arrow) appears to be originating from right coronary sinus and is a small vessel (1.7 mm at its greatest diameter). Small left circumflex artery (gray arrow) is also seen in the image posterior to the aorta. (B) A volume rendered 3D CT angiogram (posterior view) demonstrating origin of left anterior descending artery (black arrow), left circumflex artery (gray arrow) and right coronary artery (white arrow) from right coronary sinus. Small anomalous left circumflex artery has a benign course running posterior to aorta and then coming into posterior atrioventricular groove. (C) A volume rendered 3D CT angiogram (anterior view including right ventricle and pulmonary arteries) shows a malignant course of left anterior descending artery (black arrow) running between ascending aorta and pulmonary trunk.

A 22-year-old Caucasian male with no significant past medical history was evaluated in cardiology clinic with intermittent chest pain. Chest pain was nonexertional, located in the center of chest and nonradiating. The patient was a college athlete and denied symptoms of chest pain, palpitations, dizziness, or syncope with exertion. He had exercise nuclear stress test one month ago for similar chest pain which was normal. He denied personal history of heart problems or family history of premature coronary artery disease, inherited arrhythmias, or sudden cardiac death. Electrocardiogram (EKG) showed normal sinus rhythm with no ST or T wave changes suggestive of ischemia and three sets of troponin I were normal. Transthoracic echocardiogram showed normal ejection fraction of 60%-65% and no segmental wall motion or valvular abnormalities. He underwent coronary computed tomography (CT) angiogram (CCTA) which revealed large dominant right coronary artery (RCA) and anomalous origins of left anterior descending artery (LAD) and left circumflex artery (LCX) from right coronary sinus (Figures 1-2). LAD had a malignant course between aorta and pulmonary artery.

The patient underwent left heart catheterization which showed a very large dominant RCA and small LAD and LCX with anomalous origin from right coronary sinus (Videos 1-2). The coronary arteries appeared angiographically normal.

**Video 1:** Coronary angiogram shows anomalous origin of small left anterior descending artery from right coronary sinus that courses anteriorly and provides circulation to the base and mid portion of the anterior wall. No plaque was appreciated on angiogram.

**Video 2:** Coronary angiogram shows a large dominant right coronary artery. Posterior descending artery wraps around the apex. The posterior ventricular branch is large and actually wraps all the way onto anterior surface of left ventricle. There is faint filling of additional vessel likely left circumflex artery coming high off of the right coronary sinus which is not fully visualized.

**Figure 3:**
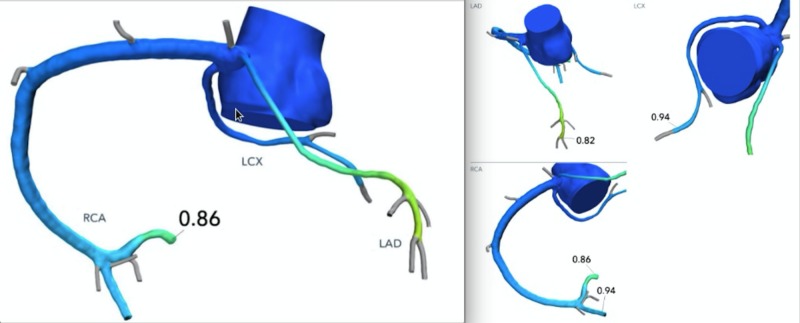
Coronary computed tomography (CT) angiography derived fractional flow reserve (CT-FFR) measurements are normal for all three vessels.

Because of chest pain and anomalous LAD with malignant course, it was decided to get noninvasive fractional flow reserve (FFR) assessment from coronary CT angiography which was hemodynamically nonsignificant (Figure 3). Based on FFR findings and small size of the vessel, it was decided to treat the patient conservatively. The patient’s chest pain was considered atypical which resolved on its own. He was recommended to continue his regular physical activities with no restriction.

## Discussion

Coronary anomalies are a rare group of congenital disorders which are mostly identified incidentally on invasive or noninvasive cardiac imaging. The highest risk coronary anomaly is the origin of left main or left anterior descending artery from right coronary sinus [2]. Most of the coronary anomalies are benign without clinical significance. However, in rare cases, these anomalies can be associated with chest pain, exercise-induced syncope or pre-syncope, arrhythmias, left ventricular dysfunction, myocardial ischemia, or sudden cardiac arrest depending on the origin and course of anomalous coronary artery [1]. Anomalous course between the aorta and pulmonary artery also termed as “malignant course” poses the greatest risk of major adverse cardiac event [3]. Coronary anomalies are one of the leading causes of sudden death in athletes. The proposed mechanisms leading to sudden death are compression of coronary artery due to slit-like orifice, vasospasm, and ventricular tachycardia [3]. Origin of anomalous coronary artery is visualized well on angiography, however, its course is best delineated by multidetector computed tomography (MDCT) [4]. Surgical repair is recommended (class 1 indication) if left coronary artery arises from right coronary sinus and have documented evidence of myocardial ischemia due to coronary compression according to ACC/AHA 2018 guidelines for the management of adults with congenital heart disease [4]. This highlights the importance of performing further testing to document ischemia for guiding further therapy. Various diagnostic tests including treadmill EKG stress test, nuclear stress test, intravascular ultrasound (IVUS), and coronary fractional flow reserve can be used for reversible ischemia testing, however, no randomized control trials comparing these tests are available [2-4]. Noninvasive fractional flow reserve with coronary CT angiography provides accurate anatomic and functional assessment and has been increasingly used recently to diagnose hemodynamically significant obstructive coronary lesions thus guiding further therapy [5]. However, its use in congenital coronary anomalies has not been studied. There have been few reported case reports of CT-FFR use in patients with coronary anomalies [6-7]. 

## Conclusions

Objective evidence of ischemia is one of the most important factors in making decision about surgical repair in coronary anomalies. Noninvasive FFR derived from coronary CT angiography can be a useful additional tool in guiding management strategy. Further studies are needed to evaluate the use of coronary CT-FFR in patients with coronary anomalies.
